# Removal of *Escherichia coli* and Faecal Coliforms from Surface Water and Groundwater by Household Water Treatment Devices/Systems: A Sustainable Solution for Improving Water Quality in Rural Communities of the Southern African Development Community Region

**DOI:** 10.3390/ijerph9010139

**Published:** 2012-01-04

**Authors:** Jocelyne K. Mwabi, Bhekie B. Mamba, Maggy N. B. Momba

**Affiliations:** 1 Department of Environmental, Water and Earth Sciences, Tshwane University of Technology, 175 Nelson Mandela Drive, Pretoria 0002, South Africa; Email: jocelyne.mwabi@gmail.com; 2 Department of Chemical Technology, University of Johannesburg, P.O. Box 17011, Doornfontein 2028, South Africa; Email: bmamba@uj.ac.za

**Keywords:** bacterial removal, household water treatment, turbidity, water quality

## Abstract

There is significant evidence that household water treatment devices/systems (HWTS) are capable of dramatically improving microbially contaminated water quality. The purpose of this study was to examine five filters [(biosand filter-standard (BSF-S); biosand filter-zeolite (BSF-Z); bucket filter (BF); ceramic candle filter (CCF); and silver-impregnated porous pot (SIPP)] and evaluate their ability to improve the quality of drinking water at the household level. These HWTS were manufactured in the workshop of the Tshwane University of Technology and evaluated for efficiency to remove turbidity, faecal coliforms and *Escherichia coli* from multiple water source samples, using standard methods. The flow rates ranged from 0.05 L/h to 2.49 L/h for SIPP, 1 L/h to 4 L/h for CCF, 0.81 L/h to 6.84 L/h for BSF-S, 1.74 L/h to 19.2 L/h and 106.5 L/h to 160.5 L/h for BF The turbidity of the raw water samples ranged between 2.17 and 40.4 NTU. The average turbidity obtained after filtration ranged from 0.6 to 8 NTU (BSF-S), 1 to 4 NTU (BSF-Z), 2 to 11 NTU (BF), and from 0.6 to 7 NTU (CCF) and 0.7 to 1 NTU for SIPP. The BSF-S, BSF-Z and CCF removed 2 to 4 log_10_ (99% to 100%) of coliform bacteria, while the BF removed 1 to 3 log (90% to 99.9%) of these bacteria. The performance of the SIPP in removing turbidity and indicator bacteria (>5 log_10_, 100%) was significantly higher compared to that of the other HWTS (*p* < 0.05). The findings of this study indicate that the SIPP can be an effective and sustainable HWTS for the Southern African Development Community (SADC) rural communities, as it removed the total concentration of bacteria from test water, can be manufactured using locally available materials, and is easy to operate and to maintain.

## 1. Introduction

Waterborne diseases have a negative impact on public health in developing countries where the drinking water is of a poor quality [1]. The *United Nations World Water Development Report 3: Water in a Changing World* [2] points out that up to 50% of malnutrition cases reported in Africa are related to recurring diarrhoea or intestinal nematode infections resulting from the consumption of contaminated water. Therefore, one of the most important prerequisites for improving the health of the people living in developing countries is the provision of safe and clean water to communities. 

In South Africa, the commitment of the Government to improve access to safe water supply is evidenced by the marked reduction in the number of people who do not have access to a reliable source of safe drinking water, from over 15 million people in 1994 down to some six million people in 2008 [3]. However, studies have shown that poverty-stricken rural communities are still dependent on unsafe drinking water containing a high rate of pathogens that cause endemic gastrointestinal disease [4,5,6]. Consequently, the morbidity and mortality rates due to water-related diseases between 2001 and 2005 were reported as being 667,300 and 92,000 for cholera, 151,000 and 6000 for typhoid fever, 35,000 and 2000 for paratyphoid fever and 112,000 and 1000 for the hepatitis A virus, respectively, *per annum* [7]. Although there are indications of a downward trend, these diseases are still persistent, with periodic outbreaks. Because of the impact of drinking water of poor microbiological quality on the health of rural communities, and especially on immunocompromised people, children and elderly people [8], there is a great need to develop or identify alternative systems by means of which people in rural communities can obtain access to safe drinking water. These alternative systems of providing safe water need to be affordable to the poorest of the poor, easy to operate, easy to maintain, and should be socially acceptable or culturally appropriate to ensure that people will adopt them, continue to use them and thus always have access to safe water [9,10]. 

Various household water treatment devices/systems (HWTS) have been developed over the years to treat water at point-of-use (POU) at the household level. Many of these devices are currently being used in various developing countries around the world as cost-effective systems for treating microbially contaminated water sources in order to produce drinking water of an acceptable quality for domestic purposes [11,12,13]. Studies have shown that these simple and relatively inexpensive home water treatment methods can result in substantial improvements in the microbial quality of drinking water and in a reduced risk of illness and death, even in the absence of improved sanitation [9]. The most appropriate technology will depend on the situation, the quality of the raw water, the availability of the required materials and equipment, the time frame in which it is to be used, the customs, preferences and education levels of the local population and the availability of personnel to provide the necessary training and monitoring for the technology to be successfully implemented [14]. However, the variation in the effectiveness is thought to depend on the technology used, population served and local conditions [15], although more research is required to understand the impact of the different factors involved. Long-term success can be achieved by identifying and implementing approaches that will convince potential users to adopt the devices and by promoting increased and sustained use of HWTS products.

The main purpose of this study was to identify an HWTS that is simple to operate, affordable to low-income households in rural communities, very efficient in removing bacterial pathogens from polluted water, and capable of producing drinking water that complies with the *South African National Standard (SANS) 241 Drinking Water Specification* [16]. In this study, indicator coliform bacteria (faecal coliforms and *Escherichia coli),* which are used to detect the faecal contamination levels in drinking water, were considered as microbial pollutants. Five selected filters were evaluated and their flow rates, efficiency in removing target bacterial pathogens and turbidity were compared to determine the most efficient filter.

## 2. Materials and Methods

### 2.1. Selection Criteria

To achieve the objective of this study, a literature survey was conducted in order to identify and select the most used HWTS products, a matrix of the selection criteria was developed, and five types of low-cost filters that seemed to be promising for South African conditions were investigated and assessed in terms of the selection criteria. These included two kinds of biosand filter (BSF), namely the conventional or standard biosand filter (BSF-S) and the novel biosand filter with zeolite (BSF-Z), as well as the bucket filter (BF), the ceramic candle filter (CCF) and the silver-impregnated porous pot filter (SIPP). The selection and evaluation criteria are summarised in Table 1.

**Table 1:** Selection criteria for choice of HWTS and criteria for evaluation.

Selection criteria—choice of devices to be evaluated in the laboratory/field	Evaluation criteria—characteristics tested during laboratory/field work
1. Can members of rural communities afford obtaining the unit? Construction and operation cost must not exceed monthly income of an average rural household.	1. Cost (capital/running).
2. Must be representative of a number of similar systems.	2. Final water quality must comply with SANS 241.
3. Must already have been extensively evaluated.	3. Turbidity of treated water must comply with SANS 241, <1 NTU.
4. Pressure requirement must not exceed two metres.	4. Ease of operation.
5. Power requirement must not exceed energy or fuel required for basic needs of a rural household	5. Socially acceptable.
6. Robustness—durability of filter.	6. Robustness (test)
7. Safety (DWAF Regulations for New Systems).	7. Safety—ensure that the HWTS unit or device is sustainable and well managed, and minimises the health impacts on the consumer.
8. Minimum product volume to be 25 L/p·d and potable volume to be 1.8 L/p·d.	8. Storage ability and ability to deliver enough water.
9. Ease of construction and operation.	9. Extensive knowledge not required by user in rural community.

### 2.2. Preparation of Household Water Treatment Devices

#### 2.2.1. Biosand Filters (BSF)

**Figure 1:**
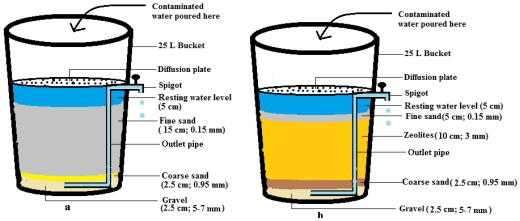
Schematic diagram of biosand filter designs: (**a**) biosand filter-standard, (**b**) biosand filter-zeolite. The figures between brackets are the diameter of grains of sand.

Two unique biosand filters were constructed for this study in the workshop of the Tshwane University of Technology, Pretoria, South Africa, based on the biosand filter developed by David Manz at the University of Calgary, Alberta, Canada, in the early 1900s, with some modifications [17]. The components of a generic biosand filter include a concrete or plastic vessel filled with filter media and gravel with elevated piping that allows the filter to maintain a 5 cm layer of water above the sand surface to prevent it from drying out. The generic size dimensions of biosand filters comprise a height of 90 cm and a width of 30 cm with the fine sand media layer being 40–50 cm high. The first modification made to the design in this study was the use of a 25 L plastic bucket (height 41 cm and width 32 cm) and filter media at a height of 15 cm. The size of the filter was scaled down to ensure that the filter would not take up too much space in a small rural home. The use of clinoptilolite zeolite to replace the fine sand of the filter media was the second modification to one of the devices during this study. The construction of the two biosand filters is described below. A schematic diagram of these two biosand filters is provided in Figure 1.

##### 2.2.1.1. Biosand Filter with Sand (BSF-S)

**Figure 2:**
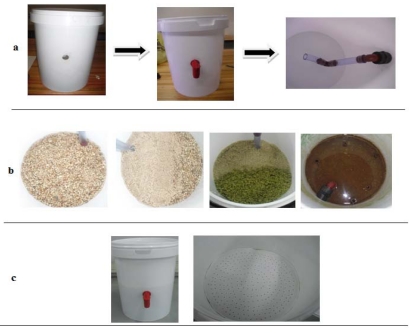
(**a**) Modifications made to buckets; (**b**) packing of sand and (**c**) final biosand filter product.

The biosand filter with sand has six distinct sections. The first section is an inlet reservoir through which contaminated water is poured into the filter. A resting water level is the second section, which was formed by raising the standpipe connected to the spigot so that a 5 cm water level would constantly be maintained above the sand surface to prevent the sand from drying out, and it facilitates the formation of the biological section by allowing oxygen to diffuse through to the biolayer. The third section is the biological layer that develops in the top 5–10 cm of sand and consists of a biofilm that develops in the top 1–2 cm of the filter sand and a layer of organic and inorganic material on the surface of the sand. This section is also known as the *Schmutzdecke*. It develops after use from one to two weeks to a couple of months as microorganisms, organic and inorganic materials from the inlet water are removed [13,17]. Its formation depends largely on the quality of the raw water treated. The fourth section consists of fine sand with a particle size of 0.15 mm, packed to a height of 15 cm. The non-biological layer forms the fifth section and is the lower region of the fine sand that contains no microorganisms due to a lack of nutrients and oxygen. The sixth section consists of coarse sand with a particle size 0.95 mm and gravel with a particle size of 5–7 mm, both packed to a height of 2.5 cm. This section supports the filter media and helps water flow into the outlet pipe without removing the sand from the filter (Figure 1a). A diffusion plate was made by perforating the lid of a 25 L bucket using a 2 mm drill bit. This was placed in the inlet reservoir above the fine sand to facilitate the even distribution of the water being filtered, to minimise the disturbance of the biological layer and to trap large suspended particles [17]. All modifications to the buckets are shown in Figure 2.

It should be mentioned that all buckets used during this study were thoroughly washed with distilled water and sterilised under a UV light for 24 h before use. The sand and gravel were also washed and rinsed thoroughly with deionised water before packing (Figure 2a,b).

##### 2.2.1.2. Biosand Filter with Zeolite (BSF-Z)

The biosand filter with zeolite was constructed in the same manner as the BSF-S described above, with an additional section consisting of clinoptilozeolite and a reduced layer of fine sand. As a number of studies have shown that zeolites have a high removal efficiency of indicator bacteria in wastewater [18,19,20], clinoptilozeolite was added to this filter to determine whether it could enhance the performance of a biosand filter. Zeolites are readily available in South Africa, are inexpensive, and should therefore be affordable to rural communities. The BSF-Z therefore has seven sections. Its components include: (1) an inlet reservoir with a diffusion plate, (2) a resting water level region, (3) a biological region consisting of (4) fine sand (particle size 0.15 mm) packed to a height of 5 cm (this thin layer of sand was included to ensure the formation of the biological layer that is crucial for the removal of pathogens), (5) a layer of clinoptilozeolite (10 cm deep and particle size 0.3 mm) followed by (6) the non-biological region which is the lower depth of the zeolite layer that contains no microorganisms or dissolved oxygen, and finally, (7) the gravel layer that was packed as mentioned for BSF-S above, which helped to support the filter media and prevent the zeolite from getting into the outlet pipe.

#### 2.2.2. Bucket Filter (BF)

**Figure 3:**
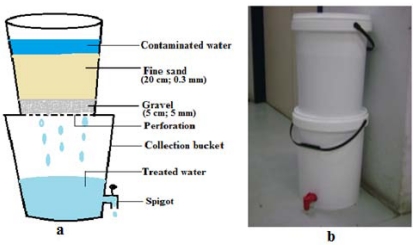
(**a**) Schematic diagram and (**b**) photograph of the bucket filter. The figures between brackets represent the thickness of the layer of sand placed in the bucket and its particle size.

The BF device was constructed from two 25 L buckets stacked on top of each other. A 20 mm hole was drilled 5 cm from the base of the bottom bucket, into which a tap was fixed for the collection of filtered water. The filter media, consisting of a 20 cm layer of fine sand (0.3 mm) and a 5 cm layer of gravel (5 mm), were packed into the top bucket. The filtering process in the BF takes place as follows: Contaminated water is poured into the upper bucket, passes through the filter media (fine sand), and the treated water accumulates in the lower bucket through the perforations in the base of the top bucket. Filtration occurs through the thick layer of fine sand while the gravel prevents the fine sand from getting into the collection vessel through the perforations [21] (Figure 3).

#### 2.2.3. Ceramic Candle Filter (CCF)

**Figure 4:**
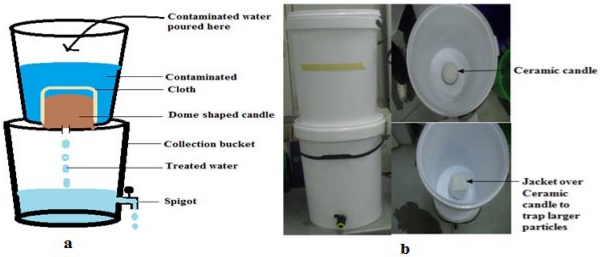
(**a**) Schematic diagram and (**b**) photograph of the ceramic candle filter.

A ceramic candle filter (a hollow, dome-shaped cylinder, 10 cm high with a diameter of 10 cm) was obtained from Headstream Water Holdings (South Africa). It was wedged between two 25 L buckets by screwing the candle to the bottom of the upper bucket through the lid of the lower bucket, and a spigot was inserted 5 cm from the base of the lower bucket, as illustrated in Figure 4. The candle filter element was covered with a thick cloth to reduce the turbidity of the contaminated water and to trap debris (leaves and insects). Contaminated water is poured into the upper bucket and is filtered through the micropores (0.2 microns) of the dome-shaped candle filter. The filtered water accumulates in the lower bucket, where it is temporarily stored until use. 

#### 2.2.4. Silver-Impregnated Porous Pot Filter (SIPP)

**Figure 5:**
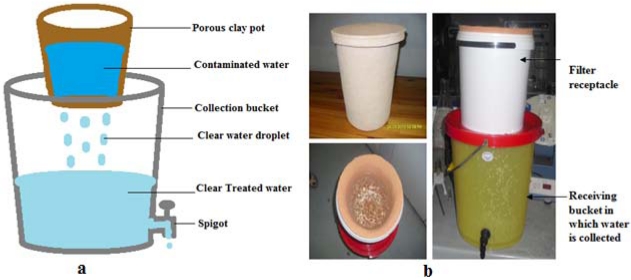
(**a**) Schematic diagram and (**b**) photograph of silver-impregnated porous pot filter.

The silver-impregnated porous pot filter (Figure 5) used in this study was developed by the Tshwane University Water Research Group (Pretoria, South Africa) as part of a previous project of the Water Research Commission of South Africa (WRC Project No. K8/810). A mixture of ball clay, sawdust, paper fibre and silver nitrate solution (23.5 g) was moulded into a pot shape. The SIPP is similar to the ceramic silver-impregnated pot filter (CSF) described by Lantagne and co-workers [22]. However, the fabrication process the SIPP was modified to include AgNO_3_ in the clay mixture prior to initial firing, instead of being coated with a 2 mL solution of colloidal silver (3.2% in 250 mL water) after firing, as described by Lantagne and co-workers [22]. The silver nitrate acts as a disinfectant due to the bacteriostatic properties of the nano-silver particles [23]. The complete water treatment system consists of the pot filter (maximum capacity 5–6 L) that is contained in a 10 L plastic receptacle (height 24 cm, diameter 26 cm) (Figure 5a,b), which is positioned on top of a 20 L collection bucket (height 33.5 cm, diameter 32 cm). The contaminated water is poured into the clay pot and slowly drips through the fine pores of the clay element into the collection vessel. A small spigot is used to withdraw water for drinking and to prevent the contamination of filtered water by dirty hands or utensils with which water is drawn (Figure 5b).

#### 2.2.5. Manufacturing Costs of Household Water Treatment Devices

With the exception of the CCF, all the materials used in the construction of the devices were purchased locally. Table 2 illustrates the total cost of the materials used during the manufacturing process. The total manufacturing cost of the CCF amounted to R 501.15 (USD 64), while the total price of a complete SIPP filter is between R 240.56 (USD 30) and R 290.56 (USD 36). The following materials were used in the construction of the biological sand filters: two 25 L plastic buckets (R 25.58 each, USD 3.2), two spigots (R49.99, USD 6.2), 1 m clear tubing (R 24.99, USD 3.1), two insert elbows (R 3.79 each, USD 0.47), 1 thread tape (R 6.49, USD 0.81), 40 kg fine sand (R 28.00, USD 3.5), 40 kg gravel (R 30.24, USD 3.8), 40 kg coarse sand (R 34.82, USD 4.4) and 50 kg zeolites (R 155.35, USD 19.4). The clear tubing and the thread tape could be used to construct two filters, the fine sand 20 filters, gravel and coarse sand 40 filters, and zeolites five filters. This resulted in the cost of the BSF-S being R 133.16 (USD 16) and the BSF-Z being R 164.23 (< USD 20.00).

**Table 2:** Total manufacturing costs of household water treatment devices.

HWTS devices	Rands (ZAR)	Dollars (USD)
BSF-S	133.16	16
BSF-Z	164.23	20
BF	149.18	19
CCF	501.15	64
SIPP	290.56	36

### 2.3. Evaluation of Performance of HWTS Devices in Improving Water Quality

The performance of the devices in removing bacteria from contaminated water performed using synthetic and environmental water sources. 

#### 2.3.1. Evaluation with Synthetic Water

The selected devices were subjected to five trials in the laboratory for the removal of *E. coli* spiked into sterile saline deionised water. *Escherichia coli* ATCC 29552 was obtained from the American Type Culture Collection (Quantum Biotechnologies, RSA) and was maintained on nutrient agar (Biolab, South Africa) plates and incubated at 36 ± 1 °C for 24 h. One loop full of this bacterial culture was inoculated into 100 mL sterile nutrient broth (Biolab, South Africa) and incubated overnight (16 h) at 37 °C in a shaking incubator (Scientific Model 353, Lasec, South Africa) at a speed of 100 ± 10 rpm, after which 1 mL of the overnight culture was serially diluted in 9 mL sterile physiological water (0.9% w/v NaCl) and spread-plated onto chromocult coliform (CCA) agar (Merck) plates. The plates were incubated at 37 °C for 24 h and the resulting colonies were counted to express the initial bacterial concentrations as cfu/mL [24]. Aliquots of the *E. coli* culture corresponding to10^6^ cfu/mL were inoculated into 20 L (final volume) of sterile saline water (0.9%). The spiked water samples were shaken vigorously several times before being passed through the HWTS. Five trials were performed to evaluate the performance of these devices using synthetic water.

#### 2.3.2. Evaluation with Environmental Water Sources

*Sampling of water sources—*Source water samples were collected from four different sites in Gauteng Province, between 27 September 2010 and 18 March 2011. Samples of surface water with a low (SWL) and high (SWH) turbidity were collected from the Apies River (Pretoria, Gauteng Province, South Africa) and from the Hartbeespoort Dam (Hartbeespoort, North West Province, South Africa), respectively. Samples of groundwater with a low (GWL) and high (GWH) turbidity were collected from boreholes in Delmas (on the border of the Mpumalanga and Gauteng provinces, South Africa) and Wallmannsthal (Gauteng Province), respectively. For this study, the water turbidity levels were categorised as 2 NTU to 18 NTU, 10 NTU to 40 NTU, 2 NTU to 10 NTU and 2 NTU to 15 NTU for the abovementioned test water sources, SWL, SWH, GWL and GWH, respectively. Test water samples were collected six times from each source in sterile 20 L plastic buckets. Water samples were also collected in sterile 1 L glass bottles in order to detect and enumerate the initial concentration of bacteria before treatment. The samples were transported to the laboratory within 6 h and assayed for microbiological contamination and turbidity level to determine the quality of the water before and after treatment [24]. 

#### 2.3.3. Running the HWTS

Source water samples were filtered as follows through each device in the laboratory: 5 L/d for SIPP and 20 L/d for each of the remaining HWTS. Filtration was done taking into consideration that rural communities could use any available water source that is accessible to them during seasonal changes. Different volumes of filtrates were collected at 1 h intervals over the 3 h period of filtration with the assumption that enough purified water would have been produced in this period for drinking and cooking. This also allowed the researcher to establish whether significant differences in the reduction of microbial contaminants could be found at different interval times and to make the necessary recommendations in terms of safe drinking water. The total volumes of all water types filtered were 305 L and 1220 L for the SIPP and the other four devices, respectively. 

*Analysis of water source quality*—Faecal coliforms and presumptive *E. coli* were determined by membrane filtration techniques using mFC agar (Merck SA) and Chromocult agar (CCA; Merck SA) plates that were incubated at 44.5 °C and 37 °C, respectively, for 24 h [24,25]. All tests were performed in triplicate. Following the enumeration, five characteristic colonies for presumptive *E. coli* were randomly selected from different plates and transferred onto chromocult media by the streak-plate method and incubated at 37 °C for 24 h. The colonies were further purified by the same method at least three times using nutrient agar (Biolab, SA) before Gram staining. Oxidase tests were then performed on those colonies that were Gram negative. The API 20E kit was used for the oxidase-negative colonies and the strips were incubated at 37 °C for 24 h. The strips were then analysed and bacterial species were identified using APILAB Plus bacterial identification software (BioMérieux, Marcy l’Etoile, France). The bacterial counts were calculated and expressed in cfu/100 mL. 

Five colonies of each test bacteria presumptively identified as *E. coli* were subcultured onto selective media three times before being used for molecular identification. An overnight culture of the reference *E. coli* strain (ATCC 29552) and each presumptive *E. coli* colony was prepared in nutrient broth at 37 °C. Bacterial cells (1 mL culture) were concentrated by centrifugation at 10,000× *g* for 5 min in a microcentrifuge (Heraeus Pico 21, Thermo Scientific) and then suspended in 200 μL sterile Milli-Q water. DNA was extracted with the ZR Fungal/Bacterial DNA Kit^TM^ (ZYMO Research, Pretoria, South Africa) according to the procedures provided by the manufacturer. 

Molecular identification of *E. coli* was done by amplification of the *uidA* gene that encodes for β-D-glucuronidase using primers that are specific (Table 3) for this gene in *E. coli*. The PCR amplification of the target DNA was carried out in a thermal cycler (MJ Mini^TM^ Personal Thermal Cycler, Biorad SA) using 200-μL PCR tubes and a reaction mixture volume of 50 μL. A reaction mixture was prepared, containing 20 ng of template DNA, 25 μL 2× Dream Taq^TM^ PCR master mix (10× Dream Taq^TM^ buffer, 2 μM dnTP mix and 1.25 U Dream Taq^TM^ polymerase) and a 10 μM concentration of each PCR primer (synthesised by Inqaba Biotechnical Industries (Pty) Ltd, Pretoria, South Africa) and was made up to 50 μL with ultra-pure nuclease-free water. The following cycling parameters were used: denaturation of template DNA at 94 °C for 2 min, followed by 25 cycles of denaturation at 94 °C for 1 min, annealing of template DNA at 58 °C for 1 min and an extension time of 1 min at 72 °C for the primers. After the last cycle the samples were kept at 72 °C for 2 min to complete the synthesis of all strands [26,27]. 

**Table 3:** PCR oligonucleotide primers used to detect *E.coli*.

Bacterium	Gene target	Amplicon size (bp)	Primer	Sequence (5′-3′)	Reference
*Escherichia coli*	*uidA*	147	UAL-754	AAAACGGCAAGAAAAAGCAG	[24]
			UAR-900	ACGCGTGGTTAACAGTCTTGCG	

An aliquot of 10 μL of PCR product was electrophoresed through a 1.5% agarose (Merck, SA) gel stained with 4.5% ethidium bromide (Merck, SA) to determine the size of the product, which was visualised under UV light in a InGenius L Gel documentation system (Syngene). A negative control and a positive control were also included in the PCR run [26]. The negative control consisted of all PCR reagents, except for the template DNA, and the positive control was the DNA of the reference *E.coli* strain at two different concentrations 10 μL and 5 μL.

#### 2.3.4. Flow Rate Testing

For the SIPP, CCF and BSF-S devices, the flow rates were measured by recording the volume of water collected from these devices after a period of 1 h. This was done over 3 h to obtain a triplicate reading. Contaminated water was passed through the HWTS devices and accumulated in the collection vessels (bottom buckets of BF, CCF and SIPP and 10 L sterilised plastic buckets for BSF-S and BSF-Z) for 1 h, after which samples (first hour collection) were taken for analysis. The collection vessel was allowed to collect filtered water for one more hour, after which the water sample was taken again for analysis (second hour collection). The third hour collection was obtained in the same manner as the first and second hour collections. The flow rate of the BSF-Z was measured by recording the volume of water collected in one minute immediately after the water had been poured into the filter; this was done at hourly intervals over a period of 3 h. A volume of 5 L was initially filtered during the first and second hour followed by filtration of the remaing 10 L in the third hour. The flow rate of the BF was measured by recording the time it took to filter 20 L of water. This was converted to L/h. Flow rate is recorded as L/h in this study.

#### 2.3.5. Turbidity Removal Efficiency

A portable turbidity meter (2100P Hach) was used to determine the level of turbidity in water samples before and after filtration. The percentage turbidity reduction achieved by each of the filter devices was calculated using the following equation:


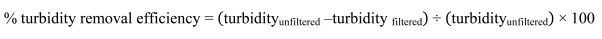


#### 2.3.6. Bacterial Removal Efficiency

The bacterial removal efficiency was obtained by comparing the concentrations of target organisms before and after treatment. Enumeration of faecal coliforms and presumptive *E. coli* after treatment was done by standard methods as mentioned above [24,25]. The Log bacterial reductions were calculated using the equation below and were converted to percentage killed [28]:


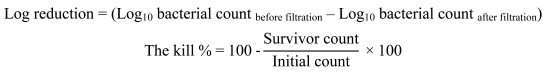


#### 2.3.7. Silver Concentration in Water Filtered by SIPP

The present part of the study is a continuous investigation on the performance of SIPP in removing pathogenic organisms from contaminated water sources. The preliminary experimental studies were conducted in our laboratory by Momba and co-authors [29,30]. During this initial study, the authors conducted a series of analytical and mechanical characterization tests on the SIPP, which included the XRF analysis that confirmed the presence of Ag, the break strength and the porosity of the filter. These authors found that the silver leached from the SIPP pot was at a level of between 0.5 and 0.6 mg/L [29], higher than the WHO recommendation of 0.1 mg/L [31]. The Ag elution was greatest in the early stages (within the first 5 L) but appeared to begin to stabilize by 10 L.

For the present study, the SIPP filter was soaked in 20 L deionised water overnight prior to use. The concentration of the silver in the filtered water was monitored at one-hour intervals over a three-hour period to determine the Ag elution by the SIPP after filtering a total volume of 305 L. The first, second and third filter runs were performed with deionised water, groundwater and surface water, respectively. The Spectro Acros ICP spectrometer (Spectro, RSA) was used to detect and determine the concentration of silver in each water sample.

#### 2.3.8. Statistical Analysis of Data

The statistical software package used to analyse the data was Stata V10 (StataCorp LP, College Station, Texas, USA). Data obtained for flow rates, turbidity and microbial contaminants after treatment were subjected to One-Way Analysis of Variance (ANOVA) to compare more than two groups. Comparisons were made between the treatment means of each device per water source to determine whether there were significant differences between treatments. Where differences were observed, pair-wise comparisons or *post hoc* tests were performed and the Wilcoxon Rank-Sum Test was used to compare the two groups. The interpretation was performed at 95% confidence limit. The tests for relationships between flow rates and turbidity or between turbidity and bacterial removal for each home water treatment were carried out using the Pearson Correlation index.

## 3. Results and Discussion

This study was unique in that the five selected devices were tested using samples of multiple water sources instead of a single water source, as reported in the literature. The objective was to determine the effect of the use of multiple water sources on the performance of the devices as well as to determine which device is the most efficient. The applicability of the HWTS to multiple water sources is important because of the differences in source water quality due to spatiotemporal and seasonal fluctuations. Therefore, a device that improves water quality and removes turbidity and bacteria under various water source conditions will provide the rural communities with high-quality water regardless of the quality of the source water, as reported by Sobsey and co-authors [13]. Twenty nine trials were performed on each HWTS: five trials using synthetic water and six trials each using the four different environmental water samples. The findings of this study are presented and discussed in the sections that follow. 

### 3.1. Flow Rates of HWTS Devices

**Figure 6:**
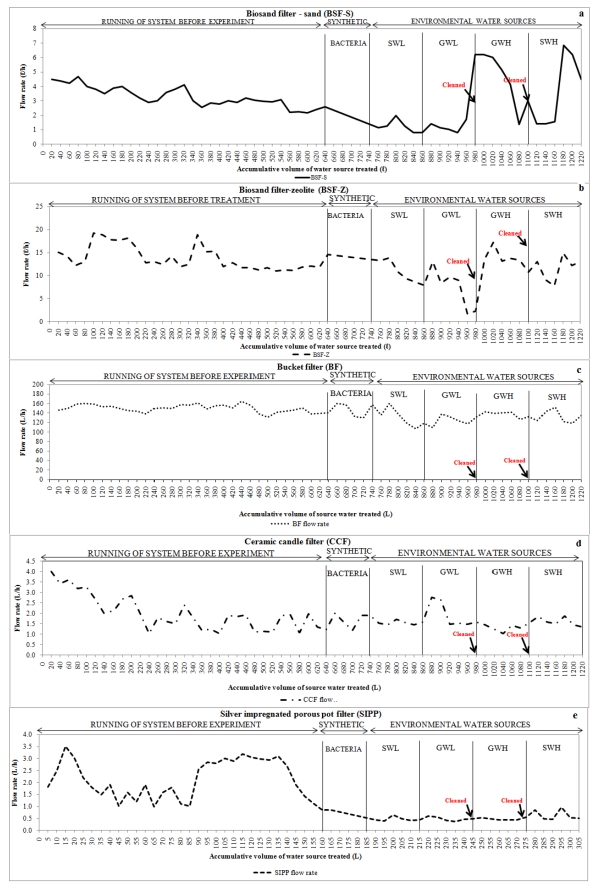
Flow rates of HWTS devices: (**a**) BSF-S, (**b**) BSF-Z, (**c**) bucket filter, (**d**) ceramic candle filter, and (**e**) silver-impregnated porous pot.

The minimum volume of potable water required per person per day for basic human activity prescribed by the Water Services Act (WSA) of South Africa is 25 L/p/d [32]. In this study, the flow rates of the selected devices were assessed to determine whether each device produced the prescribed volume of water. The results obtained showed that all devices were capable of producing 25 L/p/d, as initial flow rates averaged at 0.81–6.84 L/h, 1.74–19.2 L/h, 106.5–160.5 L/h, 1–4 L/h and 0.05–2.49 L/h for the BSF-S, BSF-Z, BF, CCF and SIPP respectively (Figure 6a–e). It was noted that the BSF-S, BSF-Z and BF could produce higher volumes than the minimum required (Figure 6a–c). However, it was observed that with increased use the SIPP and CCF ceased to produce the prescribed volume of water per day due to a decrease in the flow rate (<0.05 L/h). This gradual decline resulted from the accumulation of dirt and other particles trapped in the pores of these devices while filtering contaminated water [33,34]. A gradual decrease in the flow rates of BSF-S and BSF-Z was also observed (Figure 6a,b) as the filters matured; this was attributed to the development of the biological layer and the accumulation of dirt and other particles in the filter media [35]. In order to regain the flow rates, all devices were cleaned before filtration of groundwater with high turbidity, which was done after filtering total volumes of 980 L through each of the BSF-S, BSF-Z, BF and CCF, and 280 L through the SIPP. A drastic increase in flow rates was observed for the first three devices mentioned after the first cleaning (Figure 6a–c), whereas only a slight increase in flow rate was observed after the second cleaning process of the CCF and SIPP devices (Figure 6d–e). The BSF-S flow rate had unexpectedly increased to a value that was higher (6 L/h) than the initial flow rate (Figure 6a). The flow rate of the bucket filter was consistently higher than 100 L/h. This was due to the particle size of the media that created larger pores [34].

### 3.2. Turbidity Removal by HWTS Devices

**Figure 7:**
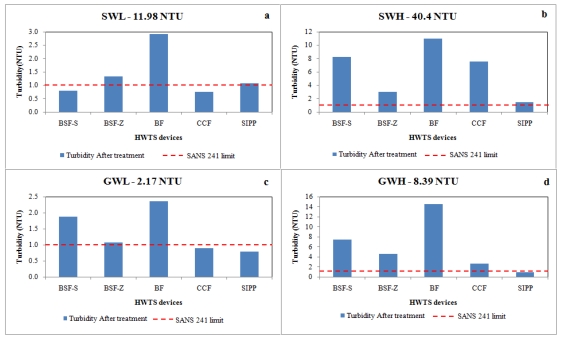
Turbidity removal by HWTS devices from each type of water source: (**a**) surface water with a low turbidity (SWL), (**b**) surface water with a high turbidity (SWH), (**c**) groundwater with a low turbidity (GWL), and (**d**) groundwater with a high turbidity (GWH).

Turbidity is a key parameter used to measure the quality of a water source [36]. The recommended limit of turbidity of drinking water by SANS 241 is <1 NTU [16] and the allowable limit is <5 NTU [37]. The average turbidity values of the water samples were found to be 11.98 NTU, 40.4 NTU, 2.17 NTU and 8.39 NTU for surface water of low turbidity, surface water of high turbidity, ground water of low turbidity and ground water of high turbidity respectively. During this study, all devices produced drinking water with turbidity levels within the allowable limit of <5 NTU, but only SIPP produced drinking water with turbidity values <1 NTU. The performance of the two biosand filters in removing turbidity improved as the filters matured (Figure 7b). This was indicated by the decrease in the average turbidity of SWH from 40.0 NTU to 2 NTU. Surface water with a high turbidity was the last and most polluted water source to be filtered. The improvement in the removal of turbidity as the biosand filter matures has been reported by Kaiser, co-authors [38], Ngai, and co-authors [39]. Particle straining is enhanced due to the development of the biological layer, and depth filtration is improved by the decrease in flow rate that increases the retention time [35,39]. The BSF-zeolite (BSF-Z) removed turbidity from all environmental water sources (except SWL) more efficiently than the Biosand- sand (BSF-S). This finding suggests that the zeolites could have played a role in removing turbidity, as this device showed 93% turbidity removal from SWH despite its high flow rate, while the BSF-S with a lower flow rate showed 80% turbidity removal (Figure 7b–d).

Strong positive correlations were found between the flow rate and the turbidity level when filtering surface water of high (SWH) and Low (GWH) turbidity and ground water of high turbidity (GWH) in the BSF-Z (*r* = 0.805), BSF-S (*r* = 0.715) and BF (*r* = 0.745), respectively (Table 4). This implies that, as the turbidity increased, the flow rate also increased and vice versa. This was unexpected, as it was assumed that, as the turbidity was removed, the flow rate would decrease due to clogging of the filter pores [33,34,39]. The flow rate of the BSF-Z was reduced as the turbidity was removed when filtering GWL, as a strong negative correlation (*r* = −0.721) was found between the flow rate and turbidity (Table 4). This finding was unexpected, as the GWL had a low turbidity (2.17 NTU). A weak negative correlation had been expected for the GWL. While a strong negative, correlation was expected between the flow rate and turbidity for the SWH, as Brown [33] and Tellen *et al*. [34] have reported that filtering a high-turbidity water source would rapidly reduce the flow rate of ceramic filters and biosand filters due to the clogging of the pores through the accumulation of solid particles. 

**Table 4:** Correlation between mean turbidity values of source water and mean flow rates of HWTS devices during filter run.

Devices	Environmental water samples (correlation coefficient: *r*)							
	SWL		SWH		GWL		GWH	
	*R*	*p*	*r*	*P*	*r*	*P*	*R*	*p*
BSF—standard	−0.376	0.0000	0.805	0.0000	0.308	0.0000	0.419	0.0000
BSF—zeolite	0.715	0.0000	0.549	0.0000	−0.721	0.0000	0.329	0.0000
BF	0.0998	0.0000	−0.0573	0.0000	0.0884	0.0000	0.745	0.0000
CCF	0.597	0.0000	0.295	0.0000	0.274	0.0000	0.398	0.0000
SIPP	0.157	0.0000	0.572	0.0000	−0.208	0.0000	−0.0300	0.0000

*r*: correlation, *p* < 0.05.

### 3.3. Bacterial Removal

The profiles of the raw water source samples used in this study were found to be unsuitable for human consumption, as the concentrations of faecal coliforms and presumptive *E. coli* exceeded the SANS 241 recommended limits for drinking water [16]. The average *E. coli* concentration in synthetic water was found to be >6.6 Log_10_, whereas the average concentrations of faecal coliforms and *E. coli* were found to be >4 Log_10_ in SWL, SWH, GWH and 3 Log_10 in_ GWL before treatment (Table 5). 

**Table 5:** Bacterial profile of source water samples (average ± SD cfu/100 mL).

Water sources	No. of trials	Organism	
		Faecal coliforms Ave ± SD	*E. coli* Ave ± SD
Synthetic water	5	-	3.68 × 10^6^ (± 3.4 × 10^5^)
SWL	6	9.4 × 10^3^ (± 9.6 ×10^2^)	2.9 × 10^3^ (± 2.8 × 10^2^)
SWH	6	7.9 × 10^3^ (± 1.27 × 10^2^)	1.3 × 10^4^ (± 8.0 × 10^3^)
GWL	6	1.4 × 10^3^ (± 1.53×10^2^)	3.0 × 10^2^ (± 3.0 × 10^1^)
GWH	6	4.0 × 10^3^ (± 5.71 × 10^2^)	5.4 × 10^3^ (± 7.24 × 10^2^)
SANS 241 (2006) [16]		0 cfu/100 mL	0 cfu/100 mL
WHO (2006 [30]		0 cfu/100 mL	0 cfu/100 mL

-: Was not spiked into synthetic water.

**Figure 8:**
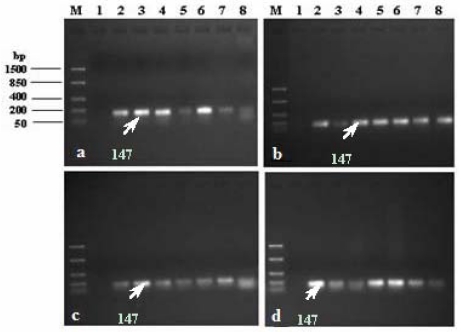
Amplification of *uidA* gene in *E. coli*. Lane M: DNA ladder, Lane 1: negative control, Lane 2: positive control: *E. coli* ATCC 25922 (10 μL), Lane 3: positive control: *E. coli* ATCC 25922 (5 μL), Lanes 4 to 8: presumptive *E. coli* colonies 1 to 5 isolated from raw water samples. Presumptive *E. coli* colonies isolated from (**a**) Apies River, (**b**) Hartbeespoort Dam, (**c**) Delmas borehole, and (**d**) Wallmannsthal borehole.

The presumptive *E. coli* colonies isolated from the various water sources were further identified using species-specific PCR techniques. The pair of oligonucleotide primers that targeted the conserved sequences of the uidA gene of *E. coli* generated a 147 bp fragment. This was an indication that the DNA of the randomly selected presumptive *E. coli* colonies and those of the positive controls *E. coli* strains, ATCC 25922, were well amplified and showed the bands located between 50 bp and 200 bp and equivalent to approximately 147 bp (Figure 8). Potable water must meet certain standards that are specified by water services authorities and public health organisations such as the World Health Organisation, as the quality of drinking water plays a major role in maintaining public health [31,37]. Thus, every effort should be made to ensure that all people have access to drinking water of a quality that is as safe as possible.

**Figure 9:**
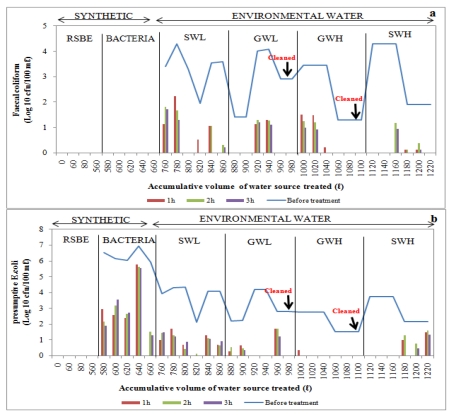
Log_10_ faecal coliform (**a**) and *E. coli* (**b**) concentrations in biosand filter-sand (BSF-S) before and after treatment of over 1220 L of source water: synthetic water and environmental water samples (SWL, GWL, GWH and SWH). RSBE is the running of the system before the experiment, using deionised water.

**Figure 10:**
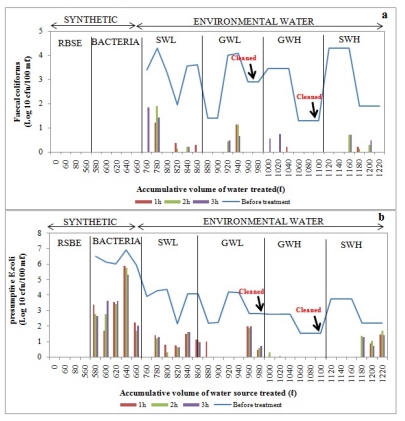
Log_10_ faecal coliform (**a**) and *E.coli* (**b**) concentrations in biosand filter-zeolite before and after treatment of over 1 220 L of source water: synthetic water and environmental water samples (SWL, GWL, GWH and SWH). RSBE is the running of the system before the experiment, using deionised water.

**Figure 11:**
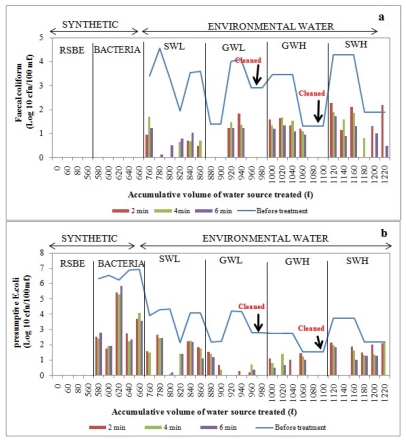
Log_10_ faecal coliform (**a**) and E.coli (**b**) concentrations in bucket filter before and after treatment of over 1220 L of source water: synthetic water and environmental water samples (SWL, GWL, GWH and SWH). RSBE is the running of the system before the experiment, using deionised water.

**Figure 12:**
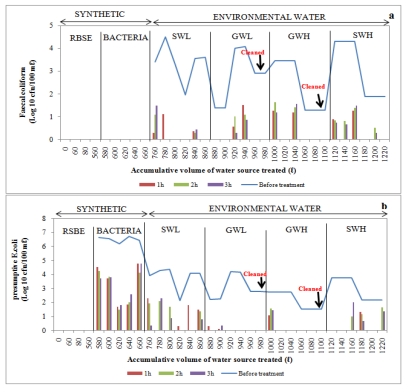
Log_10_ faecal coliform (**a**) and *E.coli* (**b**) concentrations in ceramic candle filter before and after treatment of over 1220 L of source water, namely synthetic water and environmental water samples (SWL, GWL, GWH and SWH). RSBE is the running of the system before the experiment, using deionised water.

Filtered water samples were collected at one-hour intervals over a three-hour period and the concentration of each indicator bacteria after filtration was assessed and compared to the concentration before filtration to determine the removal efficiency. Water was collected and assessed for each time interval to determine whether there was a significant difference in the removal of bacteria during each hour. In general, a considerable decline in bacterial concentrations in filtered water was noted after the filtration of synthetic water and environmental water sources, and the removal efficiency depended on the type of organism (Figure 9, Figure 10, Figure 11, Figure 12).

#### 3.3.1. Biosand Filter-Standard (BSF-S)

Generally, poor *E. coli* removal efficiency was noted when filtering synthetic water through the BSF-S, but total removal was observed in the first hour of the fifth trial (Figure 9). With surface water sources, total removal of faecal coliforms was initially noted during the third trial after filtering a volume of low-turbidity surface water (SWL) up to 800 L through the BSF-S and also within the second and third hours during the fourth trial (Figure 9a). Complete removal of *E. coli* was noted within the first and third hours after filtering up to 840 L (4th trial) of this test water source through the BSF-S (Figure 8b). Complete removal of faecal coliforms was observed during the first two trials and during the sixth trial after filtering highly turbid surface water to make up total volumes of 1120 L, 1140 L and 1220 L respectively (Figure 9a). No *E. coli* were detected during the first three treatment trials (Figure 9b).

Filtration of low-turbidity groundwater (GWL) up to total volumes of 880 L, 900 L, 960 L and 980 L using the BSF-S device resulted in the complete removal of faecal coliforms during the first two trials and also during the fourth and sixth trials, respectively (Figure 9a). There was a complete removal of *E. coli* during the third, fourth and sixth trials after filtering up to 920 L, 940 L and 960 L of GWL, respectively (Figure 9b). Total removal of faecal coliforms occurred during the last three trials after filtering highly turbid groundwater (GWH), corresponding to total filtered volumes of 1060 L, 1080 L and 1100 L, and also in the second and third hours after filtering a total volume of 1040 L (Figure 9a). No *E .coli* concentrations were noted in any of the trials, except for the first hour of the first trial (Figure 9b).

#### 3.3.2. Bacterial Removal by Biosand Filter-Zeolite (BSF-Z)

Poor removal efficiency was observed when filtering synthetic water source through the BSF-Z, as *E. coli* concentrations after treatment ranged between 2 Log_10_ and 6 Log_10_. However, complete removal of *E. coli* and faecal coliforms was observed during the first and third trials, respectively, after filtering SWL of total volumes of 800 L and 760 L (Figure 10a,b). During the filtration of highly turbid surface water, no faecal coliforms were detected during the first, second and sixth trials after filtering total volumes of 1120 L, 1140 L and 1220 L, respectively, while *E. coli* were completely removed during the first three trials (Figure 10a,b). 

Trials with low-turbidity groundwater sources indicated no faecal coliforms during the first two and last two trials after adding total volumes of 880 L, 900 L and 960 L, 980 L, respectively, to the BSF-Z (Figure 10a). Total removal of *E. coli* was noted during the second, third and fourth trials after filtering total volumes of 900 L, 920 L and 940 L of low-turbidity groundwater, respectively, as well as in the second and third hours of the first trial (Figure 10b). Adding up to 1060 L, 1080 L and 1100 L of highly turbid groundwater to the filter resulted in the total removal of faecal coliforms during the last three trials. As indicated in Figure 10a, this bacterial group could also not be detected during specific hours of the first three trials after filtration of up to 1000 L, 1020 L and 1040 L. No *E. coli* were detected during the last four trials after filtering total volumes of 1040 L, 1060 L, 1080 L and 1100 L, as well as during the second and third hours of the first two trials (Figure 10b).

In general, the trend in the removal of target bacterial pathogens in BSF-S and BSF-Z was towards increased bacterial removal with repeated filtration of raw water sources. This shows that either the development of the biological layer (*Schmutzdecke*) or the increase in the retention time with the filter media as the filtration rate decreases due to head-loss accumulation has an impact on bacterial removal [40,41,42,43]. Studies by Kaiser *et al*. [38] and Campos and his colleagues [40] have also shown that bacterial removal efficiency improves with repeated filter use and increasing time of filtration, due to filter maturation.

#### 3.3.3. Bucket Filter (BF)

The removal of *E. coli* from synthetic water source by BF was found to be poor, as can be seen in Figure 11. With surface water sources, total removal of faecal coliforms was observed in the second and fourth hours of the second and third trials after filtering total volumes of 780 L and 800 L, respectively (Figure 11a). No *E. coli* were detected during the second and third trials after filtering up to 800 L and 820 L. The complete removal of *E. coli* and faecal coliforms was achieved during the second trial and in the first and third hours of the fourth trial, respectively, after filtering total volumes of 1140 L and 1180 L (Figure 11a,b). 

During the first two trials and last two trials with low-turbidity groundwater, there was total removal of faecal coliforms after filtering up to 880 L, 900 L and 960 L, 980 L respectively of this water source through the bucket filter (Figure 11a). Complete removal of *E. coli* was observed during the fourth and sixth trials after filtering up to total volumes of 920 L and 980 L, respectively. No *E. coli* were detected in the second and third hours of the fourth trial (Figure 11b). During the treatment of highly turbid groundwater, faecal coliforms and *E. coli* were totally removed during the last two trials after filtering total volumes of 1080 and 1100 L (Figure 11a,b).

Overall, the bucket filter showed the poorest removal of faecal coliforms [2–3 log_10_ (99–99.9%)] and *E. coli* [1–3 log_10_ (90–99.9%)], as no biological layer develops on the top surface of the sand bed in a rapid sand filter to enhance the removal of bacterial pathogens (Table 6). The bucket filter does not have a resting water level as the biological sand filters have, and dries up. Thus, conditions in a bucket filter are not favourable for the development of a biological layer [9,34]. This clearly explains its poor performance in bacterial removals compared to the biosand filters. As Sobsey [9] reported that bucket filters are not expected to reduce bacteria by more than 90%, approximately 50% reduction was expected. However, the BF filter in this study removed upto 99.9%. The high performance of this rapid sand filter can be attributed to the particle size of sand used (0.3 mm), which is smaller than the size reported by Sobsey (1–3 mm) [9].

**Table 6:** Average percentage bacterial removal efficiency of HWTS from test water samples.

Devices	Faecal coliforms, Log_10_ cfu/100 mL removal efficiency (%) *n* = 18					
	SWL	SWH		GWL		GWH
BSF-Standard	2.7 (99.7)	3.7 (>99.9)		2.2 (99.2)		3.4 (>99.9)
BSF-Zeolite	3.0 (99.9)	>4.0 (100)		2.7 (99.7)		>3.6 (100)
BF	2.7 (99.7)	2.3 (99.3)		2.0 (99)		2.4 (99.4)
CCF	3.3 (>99.9)	3.2 (>99.9)		2.5 (99.5)		2.6 (99.6)
SIPP	>4.0 (100)	>4.0 (100)		>3.0 (100)		>3.6 (100)
**Devices**	***E. coli***					
	**Synthetic water *n* = 15**	**Environmental water samples *n* = 18**				
		**SWL**	**SWH**		**GWL**	**GWH**
BSF-Standard	3.6 (99.9)	2.4 (99.4)	3.1 (>99.9)		1.6 (96)	>3.7 (100)
BSF-Zeolite	3.2 (>99.9)	2.4 (99.4)	3.0 (99.9)		1.3 (93)	>3.7 (100)
BF	3.3 (>99.9)	1.5 (95)	2.2 (99.2)		1.8 (98)	3.0 (99.9)
CCF	3.2 (>99.9)	1.8 (98)	3.2 (>99.9)		>2.5 (99.5)	2.9 (99.8)
SIPP	>6.6 (100)	>3.5 (100)	>4.0 (100)		>2.5 (100)	>3.7 (100)

#### 3.3.4. Ceramic Candle Filter (CCF)

While poor *E. coli* removal efficiency was observed during filtration of synthetic water through the CCF (Figure 12), no faecal coliforms were observed during the 3rd, 4th and 6th trials after filtering low-turbidity surface water through this CCF to make up total volumes of 800 L, 820 L and 860 L, respectively. As illustrated in Figure 12a, faecal coliforms could also not be detected during the second and third hours of the second trial after filtration of up to 780 L. Filtration of total volumes of 820 L and 840 L low-turbidity surface water during the fourth and fifth trials resulted in the complete removal of *E. coli* after the second and third hours of treatment. With high-turbidity surface water, no faecal coliforms could be detected during the first two trials and also during the fifth trial after filtering up to 1120 L, 1140 L and 1200 L of water respectively in total (Figure 12b).

With groundwater sources, complete removal of faecal coliforms was achieved during the first two trials and during the last two trials after filtering up to total volumes of 800 L, 900 L, 960 L and 980 L of low-turbidity groundwater, respectively (Figure 12a). During the last four trials, no *E. coli* were observed in the filtered water samples (Figure 12b). Faecal coliform bacteria were not observed during the last three trials after filtering up to 1080 L, 1080 L and 1100 L intake GWH source (Figure 12a). With the exception of the first hour of the first trial, no *E. coli* were detected after treating this type of water source using the CCF device (Figure 12b).

#### 3.3.5. Silver-Impregnated Porous Pot (SIPP)

This device consistently produced high-quality water that did not contain any traces of faecal coliform *or E. coli* during each hourly interval for each day of the filter run throughout the study period (Table 7). The device removed between 2.5 Log_10_ and 6.6 Log_10_ of these coliform bacteria from contaminated water. The results for SIPP are presented in Table 7, as no bacteria were detected in any of the treated water samples.

**Table 7:** Average bacterial removal efficiency by SIPP from various water samples.

Water source	Number of trials	Bacterial species	Log_10_ before treatment	Log_10_ removal
Synthetic water	5	*E. coli*	6.6 Log	>6.6 Log
SWL	6	*E. coli*	4.1 Log	>4.1 Log
		Faecal coliforms	3.9 Log	>3.9 Log
SWH	6	*E. coli*	3.5 Log	>3.5 Log
		Faecal coliforms	4.0 Log	>4.0 Log
GWL	6	*E. coli*	3.7 Log	>3.7 Log
		Faecal coliforms	3.6 Log	>3.6 Log
GWH	6	*E. coli*	2.5 Log	>2.5 Log
		Faecal coliforms	3.1 Log	>3.1 Log

The average faecal coliform and *E. coli* removal and percentage removal efficiency by HWTS devices from the various water sources are summarised in Table 6. The SIPP was the only device that consistently produced drinking water complying with the recommended limits set by the *South African National Standard (SANS) 241 Drinking Water Specification* [16], regardless of the quality of the source water treated. The percentage removal of *E. coli* observed in the other four devices when treating synthetic water was >99.9%. The removal of target bacteria from the environmental water samples ranged between 2–4 Log_10_ (99.9% to >99.99%), 1–4 Log_10_ (90% to >99.99%), 1–3 Log_10_ (90% to 99.9%) and 2–3 Log_10_ (90% to 99.9%) for the BSF-S, BSF-Z, BF and CCF, respectively (Table 7). All devices removed *E. coli* within similar ranges or in some cases above the ranges reported by Elliott *et al*. [35], Baumgartner [42] and Laurent [43]. Biosand filters have been reported to remove *E. coli* in the rate ranging between 85% and 98% [35,40,41]. Chemically modified rapid sand filters may remove between 80% and 95% [43]. The removal of bacteria by the SIPP and CCF was observed to be >99% for the duration of the study. The performance of these two filters was within the ranges reported by previous investigators. The ceramic candle filter and silver-coated ceramic pot filters have been shown to remove *E. coli* in ranges between 80% and 99%, and between 95% and 100%, respectively [44,45]. 

As stated above, the preliminary investigation on the performance of SIPP in removing pathogenic organisms from synthetic water sources was conducted in our laboratory by Momba and co-authors [29]. *Escherichia coli* spiked in synthetic water were used as a prototype organism and the authors included a control porous pot that was not impregnated with AgNO_3_. It was revealed that only the Ag impregnated pot was significantly effective in removing this organism [30]. The more efficient bacterial performance of the SIPP as compared to the control porous pot was attributed to the imbedded Ag nanoparticles in the micropores [29]. The silver leached from the SIPP pot was at a level of between 0.5 and 0.6 mg/L, higher than the WHO recommendation of 0.1 mg/L [31]. The Ag elution was greatest in the early stages (within the first 5 L) but appeared to begin to stabilize after filtering up to 10 L of contaminated synthetic water. The SIPP, however, displayed a more gradual reduction for Ag released into the water [29]. 

Similar observations were also noted in the current study. The SIPP filter was found to be the most efficient device in removing faecal coliforms and *E. coli* from all test water source samples, as it reduced the maximum *E. coli* concentration of up to 6.6 log_10_ in spiked water to 0 cfu/100 mL (Table 6, Table 7). This corroborated the findings of Van Halem and co-workers [44], who determined that the highest possible reduction of *E. coli* by the silver-impregnated ceramic pot filter was 7 log_10_. The SIPP filter showed elevated silver concentrations in the filtrate from the first and the second run with synthetic water (Table 8). Although the concentration of the Ag in the filtrate gradually dropped to 0.22 mg/L after filtration of groundwater and surface water sources up to a total volume of 320 L, this value was still above the WHO recommended limits [31]. Though SIPP filter displayed high levels of Ag elution above the level recommended by the WHO, independent studies have indicated the relative non-toxicity of Ag and its health benefits [46]. 

**Table 8:** Silver concentration in filtered water.

Filter runs	Silver concentration in filtered water (mg/L)		
	Time intervals		
	1 h	2 h	3 h
Run 1: Deionised water	0.982	0.812	0.537
Run 2: Groundwater	0.247	0.230	0.218
Run 3: Surface water	0.240	0.256	0.280
WHO Limit [31]	0.1 mg/L		

The bacterial removal efficiency of the SIPP filter in the present study can be due to straining of bacteria through the fine pores of the clay pot [22,23]. It is also possible that the removal efficiency could have been enhanced by the AgNO_3_ that was embedded in the clay during the manufacturing process. The longer disinfection capacity of the SIPP could be attributed to the presence of the silver ion since independent studies have corroborated the effect of Ag in a water purification application, irrespective of substrate [47,48,49,50]. Silver has bacteriostatic properties and inactivates bacteria by disrupting the disulphide bonds of proteins in the cell membrane or by inhibiting DNA replication [23,44,51]. It is thus possible that the silver is contributing to the removal of bacteria. However, in future a study involving the comparison of the performance of the SIPP to a non-silver clay pot should be included to give a clear indication of whether the silver enhances the performance of the clay pot filter. Future studies should also include a long-term study whereby the concentration of silver in water filtered by SIPP is monitored on a weekly basis over 12 months or more. This would also indicate how the SIPP performs as the silver in leached out.

**Table 9:** Comparing the performance of devices.

Filter	Water source	Flow rate	Turbidity	Faecal coliforms	*E.coli*
SIPP *vs.* CCF	SWL	0.0002	0.0004	0.0000	0.0000
	SWH	0.0061	0.0000	0.0000	1.0000
	GWL	0.0000	0.0804	0.0012	0.0000
	GWH	0.0000	0.0000	0.0000	0.0012
SIPP *vs*. BSF-S	SWL	0.0000	0.5150	0.0000	0.5950
	SWH	0.0000	0.0050	1.0000	1.0000
	GWL	0.0035	0.0260	0.0340	0.8980
	GWH	0.0000	0.0000	0.9890	1.0000
SIPP *vs*. BSF-Z	SWL	0.0000	0.8720	0.2890	0.9730
	SWH	0.0000	1.0000	1.0000	1.0000
	GWL	0.0000	0.9940	1.0000	0.9930
	GWH	0.0000	0.0000	1.0000	1.0000
SIPP *vs*. BF	SWL	0.0000	0.0000	0.0010	0.0000
	SWH	0.0000	0.0000	0.0000	0.0000
	GWL	0.0000	0.0000	0.0020	0.0000
	GWH	0.0000	0.0000	0.0000	0.8410
BSF-S vs. BSF-Z	SWL	0.0000	0.0002	0.1872	0.2005
	SWH	0.0000	0.0000	0.6774	1.0000
	GWL	0.0000	0.0267	0.1391	0.0020
	GWH	0.0000	0.0000	0.5526	0.4004

Due to its highly effective performance, the SIPP was statistically compared to the other selected devices. The removal of faecal coliforms and *E. coli* by the CCF and BF was found to be significantly lower than that of the SIPP (*p* < 0.05) (Table 9). This was expected, as the first two devices lack a disinfectant that is able to inactivate bacteria at a rate comparable to that of the SIPP device. In the CCF, bacterial pathogens are removed through mechanical trapping and adsorption [45,52]. Mechanical trapping takes place in two ways. The first is through the surface filtration, whereby particles that are too large to pass through the pore are trapped on the surface of the candle. The second is through the depth filtration, where particles that penetrate the candle filter are trapped throughout the depth of the candle wall [52]. In the BF, the removal of particles, including bacteria, occurs through physical trapping with deeper penetration into the sand bed [9]. However, the microbiological quality of water filtered through the BF (rapid sand filter) has been revealed to be poor, as no biological layer develops on the top surface of the sand bed [9,53] to remove bacterial pathogens. The statistical analysis showed that both the BSF-S and BSF-Z performed similarly to the SIPP in removing the indicator bacteria from environmental water samples (*p* > 0.05). However, the experimental data indicated that SIPP was the best device overall, as the average removal of these bacteria from environmental water samples by the BSF-S and BSF-Z devices ranged between 96% to 99.9% while removal of bacteria by the SIPP was >99.99% throughout the study. The average removal of faecal coliforms ranged between 2.2 to 3 log_10_ or 99.2% to >99.9% for BSF-S and 3 to >3.7 log_10_ or 99.9% to 100% for BSF-Z, whereas the average removal of *E. coli* from raw water ranged between 1.6 to 4 log_10_ (96% to 100%) for BSF-S and between 1.3 to 4 log_10_ (93% to 100%) for BSF-Z (Table 6). 

The removal efficiencies obtained from this study are much higher than those reported by Stauber *et al*. [41] and Ngai *et al*. [53] for biosand filters, namely between 60% and 100%. This implies that the modified biosand filters BSF-S and BSF-Z are more efficient. The more efficient performance of the BSF-S can be attributed to the fine sand (0.15 mm) used which enhanced straining of particles due to the formation of finer pores as compared to conventional biosand filters [13,17]. The high performance of the BSF-Z can be a result of the antimicrobial properties of zeolites reported by Princz *et al.* [18], Kallo and Ming [19] and Widiastuti *et al*. [20]. Statistical analysis showed that there was a significant difference (*p* < 0.05) in the removal of *E. coli* from surface water of high turbidity by BSF-S and BSF-Z. The BSF-Z showed higher removal efficiency than BSF-S (Figure 9 and Figure 10). However these biosand filters performed similarly in removing *E. coli* from all groundwater samples and from surface water of low turbidity (*p* > 0.05) and faecal coliforms from all test water samples (Table 9). The removal of bacteria from raw water by biosand filters occurs by a combination of physical and biological processes [54]. The biological process involves a predation process, whereby algae and protozoa that develop on the surface of the top layer of sand within four to six weeks of filter use consume incoming bacteria; these organisms, along with slime or biofilm, comprise the *Schmutzdecke* or biological layer [41,53,54]. A great improvement in the removal of indicator bacteria was noted in both the BSF-S and BSF-Z as contaminated raw water was progressively treated. The bacterial removal efficiency was higher during filtration of surface water of high turbidity (SWH) compared to surface water of low turbidity (SWL) which was the first environmental water sample to be treated (Figure 9 and Figure 10). This can be attributed to the maturation of the biological layer that occurs with increased filter use [35,38]. The biological layer is also responsible for the decline in flow rate as the pores become clogged due to the accumulation of dirt in the sand bed. This increases the contact time between filter media and contaminated water thereby enhancing the performance of biosand filters over time [54]. Statistical analysis was also performed to compare the bacterial removal efficiency of the one-, two- and three-hour intervals of filtration for each filter. No significant differences (*p* > 0.05) were found between these hourly intervals across all five devices and the multiple water sources filtered. This finding implies that water for drinking can be collected at any time from these devices. However, it should be kept in mind that only water from the SIPP can be collected and consumed directly after filtration. The Water from the CCF, BSF-S, and BSF-Z filters would require a disinfection step to render their water safe for consumption.

Turbidity is a measure of the clarity of a liquid and was once considered mainly an aesthetic characteristic of drinking water [36,55]. However, some studies have shown that controlling turbidity is a key step in eradicating pathogens from drinking water, as microorganisms can be embedded or attached to the surface of larger suspended particles. Therefore, the removal of the larger particles could result in the removal of pathogenic microorganisms [56]. Correlations between turbidity reduction and bacterial reductions were performed to determine the relationship between the turbidity and the bacterial concentration of a water sample. The results from our study show that there is a direct relationship (as the turbidity was reduced, the indicator bacteria were also reduced) between turbidity and bacterial removal as strong positive correlations were observed between the removal of *E. coli* and turbidity from groundwater of low turbidity by the BSF-S (*r* = 0.761) and BSF-Z (*r* = 0.780). LeChevallier and Norton [55] and Fox [56] who reported that water samples with a lower turbidity are associated with lower microbial counts and vice versa substantiate these findings. However, strong negative correlations were observed between the removal of turbidity and removal of faecal coliforms and *E. coli* from SWH and GWH by the BSF-S (*r* = −0.721), BSF-Z (*r* = −0.796) and CCF (*r* = −0.784), respectively (Table 10). These findings imply that an indirect relationship existed between the turbidity and bacterial concentration (as the turbidity was reduced by these devices, the concentration of faecal coliforms or *E. coli* increased and *vice versa*). However, from the graphs it can be seen that 1–3 log (90%–99.9%) reduction of the microorganisms was achieved (Figure 9a, Figure 10a). It is possible that the turbidity value remained high even though high bacterial removal efficiencies were observed. This can be explained by the fact that organic and inorganic materials, slit, clay and soluable coloured organic particles largely contribute to the turbidity of a water source, as opposed to bacteria or viruses, which are small and translucent [55]. 

**Table 10:** Pearson’s correlation values show relationship between removal of source water turbidity and bacterial removal efficiency of HWTS devices.

**Organism**	**Devices**	**Environmental water samples (correlation coefficient: *r*)**							
		**SWL**		**SWH**		**GWL**		**GWH**	
		*R*	*p*	*R*	*P*	*r*	*p*	*R*	*p*
Faecal coliforms	BSF-S	−0.294	0.002	−0.721	0.000	−0.041	0.001	0.481	0.003
	BSF-Z	−0.044	0.043	−0.796	0.023	0.541	0.000	0.255	0.001
	BF	−0.187	0.001	0.538	0.000	−0.126	0.000	0.037	0.000
	CCF	−0.486	0.000	-	-	0.452	0.000	−0.784	0.000
	SIPP	-	-	-	-	-	-	-	-
**Organism**	**Devices**	**Environmental water samples (correlation coefficient: *r*)**							
		**SWL**		**SWL**		**SWL**		**SWL**	
		*R*	*p*	*R*	*P*	*r*	*p*	*R*	*p*
*E. coli*	BSF-S	−0.099	0.000	0.041	0.000	0.761	0.001	-	0.002
	BSF-Z	0.285	0.000	0.315	0.000	0.780	0.000	-	-
	BF	−0.102	0.000	0.328	0.000	−0.021	0.000	−0.231	0.000
	CCF	0.267	0.000	−0.453	0.000	−0.192	0.000	−0.877	0.001
	SIPP	-	-	-	-	-	-	-	-

-: No correlation was determined as no organisms were detected in the filtered water.

## 4. Conclusions and Recommendations

This study confirmed that all the selected devices are cost-effective and can improve the quality of contaminated water. However, the removal efficiency of turbidity and bacteria varied from one device to another and mostly depended on the type of water source treated. The performance of the silver-impregnated porous pot was the most efficient, as it produced drinking water that complies with the recommended limits set by the *South African National Standard (SANS) 241 Drinking Water Specification* in terms of turbidity and indicator coliform bacteria, regardless of the type of water source. Its high performance can be attributed to the bacteriostatic properties of the silver that was mixed with the clay during the manufacturing process. Unpublished data by Momba and co-authors showed that SIPP was much more efficienct at removing *E. coli* than the non-silver control clay pot filter [30]. The CCF, BSF-Z and BSF-S devices, also reduced bacteria considerably, but would require an additional disinfection step to kill residual bacteria. The performance of the CCF and BSF-S is due to the high straining efficiency attributed to the fine micropores (0.5 μm) of the ceramic shell and fine pores formed in the filter media of the BSF-S because of the fine sand. The high bacterial removal observed for the BSF-Z can be attributed to the layer of zeolite used as the filter media. Kallo and Ming [19] have previously reported that natural zeolites can remove bacterial pathogens as well as viruses. Studies by Nikashina and Mayaosedov [57] showed that up to 100% removal of faecal coliforms and *E. coli* can be achieved with zeolite that is chemically modified with amine groups-polyhexamethylene guanidine chloride (PHMG) linked with epichorohydrin. The high performance of the BSF-S and BSF-Z cannot be limited to the fine pores, zeolite and *Schumdztdecke* as various other parameters such as flow rate, temperature and type of water source can affect the performance of biosand filters [52,53]. The bucket filter was the least efficient device overall, due to the high concentration of residual bacteria in the filtered water. It is thus not advised that it be used as a household water treatment device. Alternatively, it could be used as a pretreatment filter for the other HWTS devices. It is recommended that future research be conducted on the social acceptability of these devices, as it is crucial that they not only be efficient and affordable, but also culturally acceptable to ensure that their use becomes sustainable. The best two or three devices would have to be selected and introduced to a rural community for the social aspect study. If all devices are presented to the community, the devices with the higher flow rate might be preferred by the rural users simply because of the large quantity of water produced, whereas the efficiency and effectiveness in removing pathogens should be the first priority. Future studies should also be conducted on the biosand filter containing zeolite, as this device is a novel household water treatment system. Its ability to remove turbidity and bacterial contaminants at a high flow rate shows that it has great potential to provide the required volumes of drinking water needed by rural communities for drinking and cooking. By reducing the particle size of zeolite from 3 mm to between 0.15 mm and 0.9 mm, its performance would perhaps be further improved. It is also recommended that future studies should include an experiment whereby the leaching of silver in water filtered by the SIPP is monitored on a weekly basis for the duration of the study to monitor the leaching of silver over a long period. The addition of silver to the CCF could also improve its performance. 
